# A Qualitative Study of Preschool Children’s Perspectives on an Oral Health Promotion Program in New South Wales, Australia

**DOI:** 10.3390/children11040415

**Published:** 2024-04-01

**Authors:** Jinal Shashin Parmar, Prathyusha Sanagavarapu, Sowbhagya Micheal, Navira Chandio, Susan Cartwright, Amit Arora

**Affiliations:** 1 School of Health Sciences, Western Sydney University, Campbelltown Campus, Locked Bag 1797, Penrith, NSW 2751, Australia; 18467909@student.westernsydney.edu.au (J.S.P.); 18154463@student.westernsydney.edu.au (N.C.); 2 Health Equity Laboratory, Campbelltown, NSW 2560, Australia; 3 School of Education, Western Sydney University, Bankstown Campus, Locked Bag 1797, Penrith, NSW 2751, Australia; p.sanagavarapu@westernsydney.edu.au; 4 Translational Health Research Institute, Western Sydney University, Campbelltown Campus, Locked Bag 1797, Penrith, NSW 2751, Australia; s.micheal@westernsydney.edu.au; 5 School of Medicine, Western Sydney University, Campbelltown Campus, Locked Bag 1797, Penrith, NSW 2751, Australia; 6 Colgate-Palmolive Pty Ltd., 420 George St., Sydney, NSW 2000, Australia; susan_cartwright@colpal.com; 7 Oral Health Services, Sydney Local Health District and Sydney Dental Hospital, NSW Health, Surry Hills, NSW 2010, Australia; 8 Discipline of Child and Adolescent Health, Sydney Medical School, Faculty of Medicine and Health, The University of Sydney, Westmead, NSW 2145, Australia

**Keywords:** early childhood caries, children, preschool, oral health promotion, toothbrushing, early childhood education and care setting

## Abstract

In Australia, dental caries are observed in almost half of children starting school. Oral health promotion programs are being implemented in early childhood education and care (ECEC) settings to promote oral health. This study examined children’s perceptions of one such program, the Bright Smiles Bright Futures (BSBF) program in ECEC settings in New South Wales, Australia. Data were collected using focus group discussions from 15 children aged 3–5 years, transcribed verbatim, and analysed through inductive thematic analysis. Three themes were identified as follows: (i) oral health knowledge of children, (ii) oral hygiene practices routine and skills development, and (iii) evaluation of the oral health promotion kit and opportunities for improvement. Children’s perspectives highlight the BSBF program’s success in communicating key messages to promote oral health. The integration of family-centric approaches, acknowledgement of children’s preferences, and the use of interactive tools collectively enhance the overall effectiveness of the oral health promotion program.

## 1. Introduction

In Australia, by the time children start school almost half will have experienced dental caries (tooth decay) [1], which negatively impacts their quality of life and learning outcomes [2] and places a significant burden on families, society, and the healthcare system [3]. In the most recent Australian National Child Oral Health Study (2012–2014), approximately 42% of children in Australia experienced tooth decay in their primary (baby) teeth, while 24% experienced decay in their permanent (adult) teeth [1]. Dental caries accounts for the second highest expenditure among Australian Burden of Disease Study listed conditions ($4.5 billion) [4]. Despite over a billion dollars spent on child dental care annually, child oral health in Australia has not improved since the 2000s [5]. The burden of dental caries is not unique to Australia. The Global Burden of Disease study estimated that 530 million children experienced tooth decay in their primary teeth worldwide [6]. Dental caries can have a significant impact on a child’s well-being, as it affects children’s oral health as well as their overall quality of life [7,8,9]. This can be due to a decrease in self-confidence, difficulty with socialisation, hindered learning abilities, compromised nutrition, and delayed growth [7,9].

Early childhood caries (ECC) is defined as the presence of one or more decayed (non-cavitated or cavitated lesions), missing (due to decay), or filled tooth surfaces in any primary teeth in children younger than 6 years of age [10]. ECC has a multifactorial etiology with biological, behavioral, and sociodemographic influences [8,11,12]. Evidence suggests that young children are most likely to develop ECC if *Streptococcus mutans* is acquired at an early age, although this is influenced by other factors such as poor oral hygiene, lack of regular toothbrushing with a fluoridated toothpaste, and a high-sugar diet [12]. Despite the complex etiology, it is pertinent to note that dental caries is preventable [13]. To effectively prevent dental caries in children, it is recommended to brush twice daily with an age-appropriate fluoridated toothpaste, consume a low-sugar diet, and schedule regular dental check-ups [14,15,16]. In addition, programs focused on oral health promotion have a substantial role in enhancing oral health knowledge and awareness among both children and parents [17].

The World Health Organization (WHO) launched the Global School Health Initiative in 1995 to improve the health of students, staff, families, and communities [18,19]. The focus of this initiative was to use schools as a setting to develop and support a range of healthy habits in childhood [20]. In 2003, the WHO highlighted the rationale of capitalising on schools as a setting for oral health promotion [21]. Since then, a number of oral health promotion programs have been implemented in educational settings (preschools and schools) worldwide to promote oral health [22,23]. A global scoping review by Dickson-Swift and colleagues identified several oral health promotion programs such as Top Tips for Teeth, Smiles 4 Miles, and Happy Teeth Resource Kit in Australia, and Building Brighter Smiles, Keep Smiling, and Empower elsewhere [24]. These programs are aimed at educating children on the importance of maintaining healthy teeth and serve as valuable resources to help children develop healthy habits [17,22,23] for preventing oral disease, reducing long-term health care costs, and improving quality of life [25].

Children’s involvement in general health and oral health research has gained increased attention in the last two decades [26,27]. It is now recognised that children’s perceptions of health and well-being may diverge substantially from those of adults; for instance, Statham and Chase [28] noted marked differences in how children conceptualise well-being compared to adults. Children’s active involvement in health promotion initiatives such as Health Promoting School [18] and Oral Health Promoting School [21] has also been endorsed by the WHO as a viable strategy for effective health promotion in the school environment. Despite this increased attention [27], children’s involvement in oral health research remains passive [29]. Not surprisingly, a systematic review on children’s involvement in oral health research reported that researchers did not collaborate or consult with children during intervention design, implementation, analysis, or study process [30]. Although oral health promotion interventions are effective [16,31,32,33], there is a paucity of evidence on children’s involvement in oral health promotion programs [34,35]. A recent systematic review by Chandio et al. [34] highlighted that children’s perceptions are critical for developing, implementing, and sustaining oral health promotion programs. However, their involvement and responsiveness in oral health promotion may face constraints such as inadequate engagement, insufficient communication, and misinformation transfer [34]. Nonetheless, it is pertinent to note that, while children may develop an understanding of oral health, it remains paramount for parents, caregivers, and educators to maintain a central role in health promotion by offering guidance, consistent support, and ongoing reinforcement of the significance of healthy habits.

The Bright Smiles Bright Futures (BSBF) program is an oral health promotion program launched by Colgate-Palmolive Company (New York, NY, USA) in the United States in 1991 with the aim of providing free educational resources to children in need [36]. In Australia, the BSBF program has benefited approximately 8.9 million children [36]. The program has expanded to become a global initiative, reaching over a billion children in 80 countries [36]. The BSBF program includes an interactive educational kit that includes teaching materials for educators, a brochure that children take home with instructions on how to brush their teeth, a chart to monitor their toothbrushing activity, and links to an online BSBF website with learning resources for children and parents. This study aimed to examine children’s perceptions of the BSBF program in New South Wales, Australia, and to identify necessary support for successful implementation and sustainability of the BSBF program in early childhood education and care (ECEC) settings.

## 2. Methods

### 2.1. Research Design

A qualitative study design was employed to understand children’s knowledge of oral health and their experiences and views on the usefulness of the BSBF preschool curricular activities and resources. Qualitative research is particularly suitable for understanding participant perceptions due to its open and exploratory nature, and its flexible design allows for in-depth investigation [37].

The method and reporting of results were in accordance with the Consolidated Criteria for Reporting Qualitative Research (COREQ) [38], a commonly used checklist in qualitative studies (Table S1).

### 2.2. Recruitment

Data collection utilised convenience sampling [39], recruiting children aged three to five years from preschools in New South Wales, Australia. Information on the study was sent to parents through the selected preschools, and consent was obtained before arranging children’s focus groups. The recruitment process involved emailing parents an information sheet with details on the study’s objectives, benefits, and potential risks.

### 2.3. Data Collection

Data were collected using focus groups with young children. Focus groups were chosen because they enable young children to express their views and/or share their life stories and experiences, without adhering to a structured, formal, sequenced approach in individual interviews [40]. Further, focus groups are also appropriate in research involving young children as they help to create safe spaces for their participation by addressing power imbalances which are likely to occur in one-to-one interviews [40]. The researchers ensured that the focus group’s methodology suited the developmental needs of young children. Interactive activities such as drawing and demonstration of steps in toothbrushing using a model toothbrush were included in the focus groups. Such activities are proven to be effective in engaging children in focus groups and are developmentally appropriate to collect data from young children [40,41].

During the focus group, an educator from the ECEC stayed with the children to facilitate smooth participation and minimise disruptions. The educator’s presence also helped to build children’s familiarity and rapport with the researchers [42]. Two researchers were involved in each focus group. A researcher experienced in collecting data from young children led the focus group. The second researcher, who had expertise in early childhood oral health, took notes and/or asked follow-up questions as needed. Children were seated in a circle to promote engagement by maintaining eye contact, ensuring that everyone could see and hear each other, and to minimise potential behavioural disruptions [43]. Respecting children’s right to know about the project [44], the researchers ensured that children were informed about the study, the format of the focus group, and recording procedures before starting each focus group. In total, three focus groups were conducted, each with 5–6 children [45]. Data saturation, the point where all dimensions of interest are explored and no new information seems to be added [46], was achieved after three focus groups. Guided by current literature on focus groups with young children, each focus group lasted for 20–30 min [40].

### 2.4. Data Analysis

Thematic analysis was used as it is a foundational method for qualitative analysis [47]. Data were analysed thematically to interpret the main findings of the focus group transcripts following the six-step analysis process [47]. The first step was data familiarisation, where researchers immersed themselves in the data through repeated reading of transcripts to gain a comprehensive understanding. The second step involved systematically labelling relevant features of the data and generating initial codes. Researchers then identified patterns and similarities among codes to search for themes. The fourth step comprised critically reviewing the themes to ensure data accuracy. During the fifth step, the themes were confirmed, defined, and given clear and concise names. Researchers then organised the themes into a coherent narrative that addressed the research question in the final step.

### 2.5. Methodological Rigour

To ensure rigour in the study, several methodological strategies were adopted. The children’s focus groups were conducted by researchers having extensive experience in early childhood education, population oral health, and qualitative research to ensure study credibility. To achieve verbatim transcription, an independent review, cross-checking, and coding were employed. This process ensured the accuracy and completeness of the transcription. JP, PS, and AA developed the codes. The codes were then reviewed by SM to ensure credibility and confirmability, and to facilitate conflict resolution through collective decision-making. To strengthen the rigour, a negative case analysis was undertaken [48]. This paper provides details on the methodology used making it possible to replicate the study and ensure its dependability and transferability. As part of confirmability in robust qualitative research, participant quotes are used to demonstrate data-driven findings [49].

### 2.6. Researcher Positionality

The positional attributes of researchers, encompassing elements such as age, gender, culture, and social class, wield a substantive influence on the research process [50]. To recognise the potential impact of the researchers’ positions on the study, reflexivity was employed. All researchers possessed professional and/or research backgrounds in early childhood education, dental public health, and sociology, shaping the analysis and interpretation of findings. The focus was on promoting oral health while identifying barriers and facilitators. Methodological steps were rigorously implemented to ensure objectivity, involving independent participant recruitment, the maintenance of a non-judgmental demeanour during focus groups, and the adaptation of an iterative data analysis approach to safeguard and perpetuate a stance of neutrality [50].

### 2.7. Ethical Considerations

The study, approved by the Human Research Ethics Committee at Western Sydney University (approval number H14372), involved voluntary participation. Parents received detailed information about potential risks and benefits for their children and were asked to provide written consent to allow their child to participate in the focus groups. As young children are not able to provide written informed consent, their assent was sought. Cocks [40] defines ‘assent’ as a verbal or nonverbal confirmation that children have grasped “the relationship between the researched and the researcher, trust within that relationship and acceptance of the researchers’ presence”. A detailed dialogue sheet to seek children’s assent to participate in the focus group was developed and approved by the ethics committee. Using this dialogue sheet in simple English, the researchers explained to children the purpose of the focus group, that the conversation was being recorded, and whether they agreed to participate. Children were assured that they could leave the focus group at any time.

## 3. Results

A total of 15 preschool children participated in the three focus groups. The total sample included seven boys and eight girls, aged 3–5 years.

Three major themes were identified from the data analysis: (1) oral health knowledge—implicit knowledge children have about oral health; (2) oral hygiene practices—skill development in children as part of their routine and learning from parents; (3) evaluation of the BSBF oral health promotion kit—opportunities for children to extend their knowledge about oral health and hygiene (Table 1).

### 3.1. Theme 1. Oral Health Knowledge

Children described their understanding of oral health and the rationale for taking care of their teeth. Most children were aware of the consequences of poor oral health. They demonstrated their grasp of the proper techniques and procedures necessary for maintaining a healthy mouth.

#### 3.1.1. Importance of Oral Health

**Figure 1:**
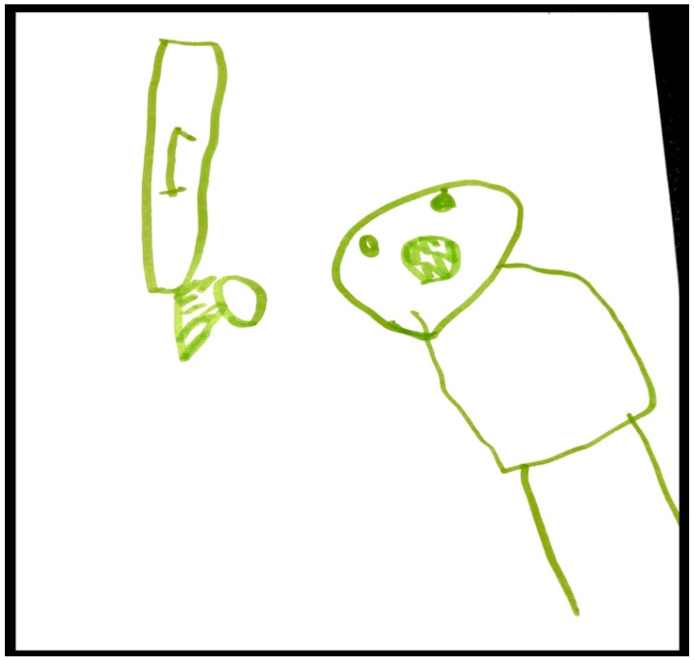
Pictorial representation of toothbrushing.

The majority of children knew it was important to maintain good oral health for eating food, smiling, and preventing teeth-related problems. Most children associated good oral health with toothbrushing every morning. A few students recognised that it was important to keep the gums and tongue clean too (Figure 1). It was evident that their knowledge of oral health care significantly impacted their toothbrushing behaviour as illustrated in the quotes below:

“*So I can eat and smile. To keep it clean. To make the gum clean with teeth also.*”

(Child 13, 4-year-old girl)

“*We brush our teeth the first thing in the morning.*”

(Child 3, 3-year-old girl)

“*We have to clean the gums and tongue too.*”

(Child 14, 4-year-old boy)

The majority of children expressed that maintaining good oral health prevented teeth-related problems. Children explained tooth loss by referring to the phrase “teeth fall off”. They described the risk of developing cavities and other health problems as “get cavity” and “makes you sick”. They expressed concern that poor oral hygiene leads to dental caries as “get black” and “makes a hole”. They effectively conveyed their understanding of concerns regarding deleterious consequences associated with suboptimal oral health, which reflected their inherent awareness of the significance of good oral health.

“*Get black cavity, we’ll get a hole, and they (teeth) fall.*”

(Child 3, 3-year-old girl)

“*Your teeth will start falling.*”

(Child 13, 4-year-old girl)

#### 3.1.2. Preventing Tooth Decay

While the majority of the children expressed their understanding of the importance of oral health, they also had a grasp of effective strategies to prevent tooth decay. They succinctly described their knowledge of the key factors for preventing tooth decay. Most children expressed the idea of limiting the consumption of sugary foods, particularly lollies, which showed their understanding that sugar intake was a risk factor for tooth decay. They also mentioned the concept of ‘moderation’, specifically when it came to consuming lollies, which indicated their understanding that enjoying lollies as a treat in moderation was acceptable. Children also discussed that cleaning their teeth after consuming lollies can lead to better oral health as shown in the quotes below:

“*We eat some lollies; it makes our teeth get holes.*”

(Child 14, 4-year-old boy)

“*You have to brush your teeth after lollies.*”

(Child 10, 4-year-old boy)

A few children shared their perspectives regarding the significant role of oral health professionals in addressing various oral health problems. Children also recognized that regular check-ups could help identify any early signs of tooth decay or other dental issues, as mentioned in the quotes below:

“*You have to go to the dentist.*”

(Child 2, 3-year-old girl)

“*You seat* [sic] *on the chair. Yeah, and then the doctor will see your mouth and gum.*”

(Child 14, 4-year-old boy)

A common view expressed by children was that taking care of their teeth was important to prevent tooth decay. Drinking tap water regularly, particularly after eating sugary foods, can help in cleaning their teeth. Children suggested that drinking tap water and eating a healthy and nutritious diet could help to prevent tooth decay. They expressed their understanding of preventative strategies in a concise yet informative way:

“*Drink lots of tap water.*”

(Child 4, 4-year-old boy)

“*Eat fresh fruits and vegetables.*”

(Child 6, 3-year-old girl)

“*Eat healthy snacks.*”

(Child 13, 4-year-old girl)

“*Don’t eat too many lollies.*”

(Child 2, 3-year-old girl)

While not explicitly mentioned in their descriptions, many children seemed to understand the importance of taking time to clean their teeth. They recognised that brushing their teeth in a rush could result in tooth decay as highlighted in the quotes below:

“*We brush our teeth slowly because we can’t go fast, we may miss one tooth.*”

(Child 3, 3-year-old girl)

“*I do slow when I do by myself when my mom and dad do, they go fast.*”

(Child 9, 3-year-old girl)

### 3.2. Theme 2: Oral Hygiene Practices

Most children demonstrated their daily toothbrushing routine with fluoridated toothpaste. In most instances, children indicated that they would brush their teeth with the help of their parents. Most children demonstrated the correct sequence of toothbrushing supported by meaningful gestures. Furthermore, a few children mentioned their mouth rinsing routine after eating.

#### 3.2.1. Making Oral Hygiene a Part of Daily Routine and the Role of Parents

**Figure 2:**
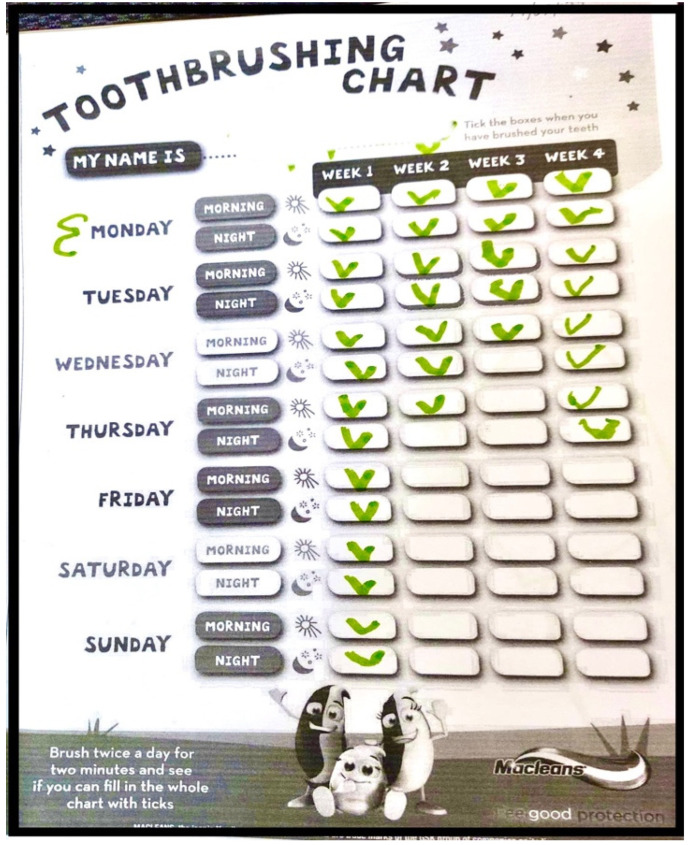

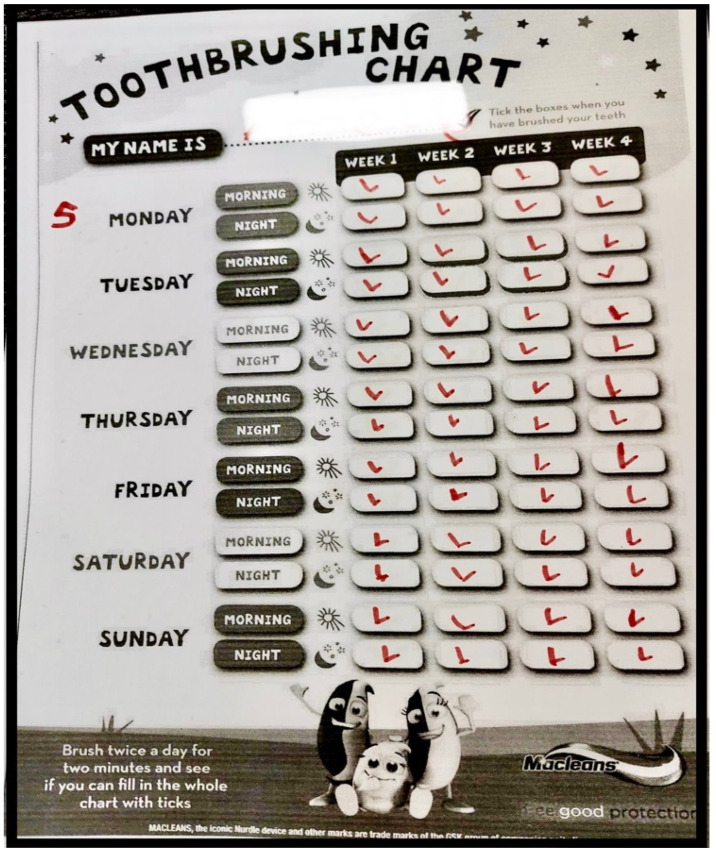
The frequency of toothbrushing in a typical week.

All children expressed their understanding of the importance of toothbrushing. The majority of children explained the simple yet impactful action of brushing their teeth every morning and night. Only a few children mentioned their routine to clean their teeth in the afternoon as well (Figure 2), as illustrated in quotes below:

“*I brush in the morning, night-time, I do in the middle of the day as well.*”

(Child 14, 4-year-old boy)

“*I brush in the morning and the night.*”

(Child 7, 5-year-old girl)

Parents play an important role in developing oral hygiene skills and routines for their children, primarily through situated learning. Children explained very briefly about their self-initiated toothbrushing efforts, coupled with guidance from their parents. This highlighted the active participation of parents in shaping their children’s oral care routine and skills. As articulated in the quotes below, children brushed their teeth by themselves, but parents ensured that they did it correctly and did not miss any area of the mouth. These findings exemplified the collaborative approach of parents and children to establish a good oral hygiene routine for their children.

“*Sometimes I do it myself, sometimes mummy daddy help.*”

(Child 1, 3-year-old girl)

“*I brush my teeth all by myself but mum checks if I am doing it correctly.*”

(Child 13, 4-year-old girl)

Children also highlighted that they acquired toothbrushing skills by observing elders (e.g., parents and older siblings) in the household. Their explanation of their learning process was succinct and emphasised the concept of situated learning. Their skill acquisition was ingrained, indicating a form of learning that took place within the context of their daily routine. This highlights the importance of role modelling and experiential learning, where children learn skills by witnessing the actions of those around them.

“*I do floss. I know it by seeing my brother.*”

(Child 12, 4-year-old girl)

“*We all brush together, I see how mom and dad do, and then I do it myself.*”

(Child 11, 4-year-old boy)

#### 3.2.2. Steps to Follow in Oral Health Care

**Figure 3:**
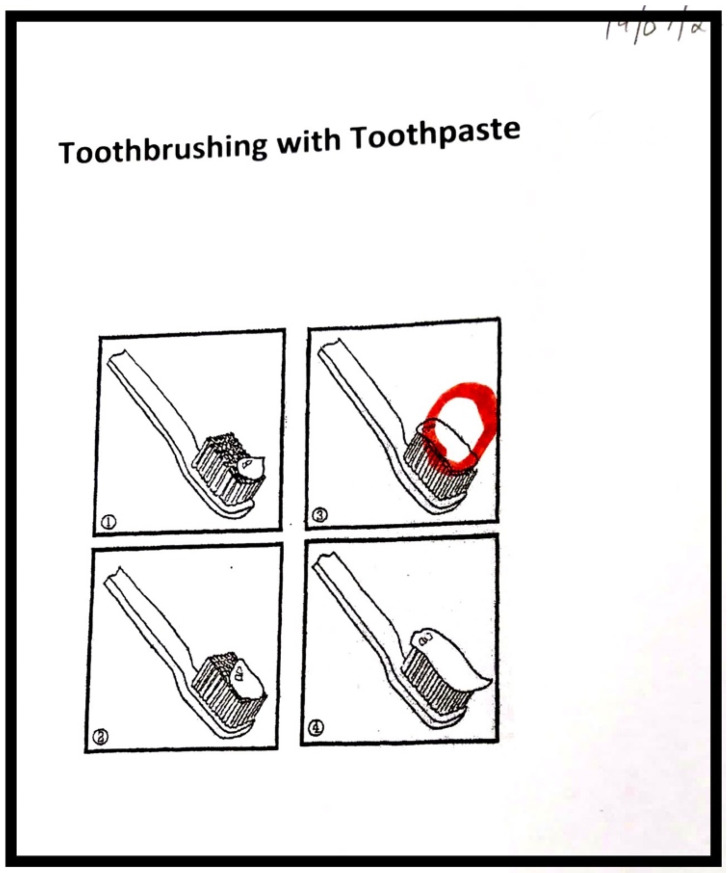

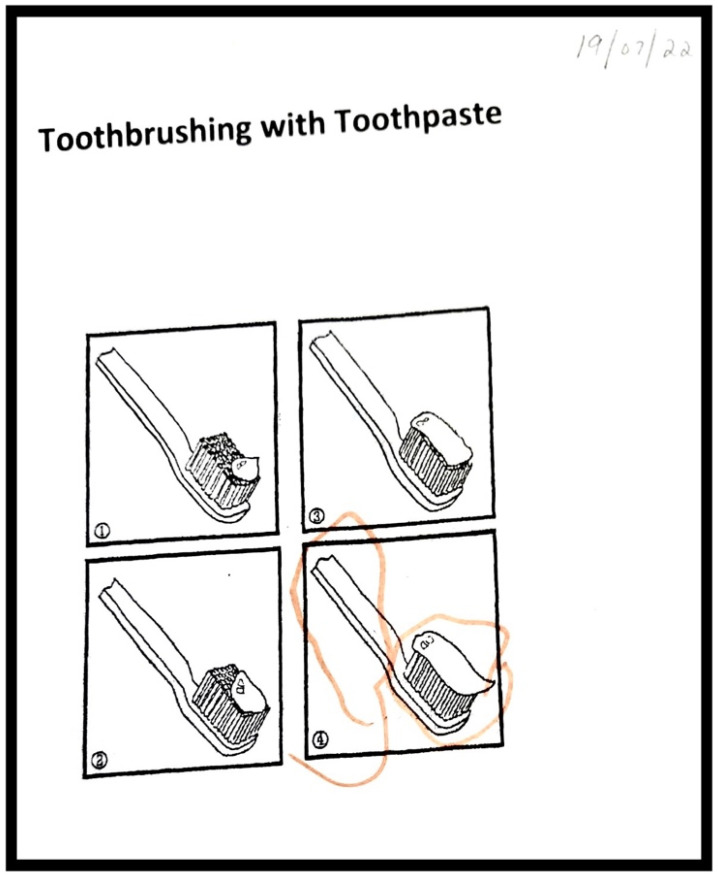
The amount of toothpaste to put on the brush.

The majority of children described the correct sequence of toothbrushing. They expressed their skill by narrating the steps of toothbrushing and providing brief descriptions of what they do, accompanied by gestures or drawings (Figure 1 and Figure 3). While they could narrate the steps involved, their limited verbal expression indicated that this knowledge was largely implicit, as exemplified in a quote below:

“*Brush the top, the inside the tongue, and put teeth together and brush, and then I do on the bottom.*”

(Child 1, 4-year-old girl)

“*Wash your hand first because we do not want to put the germs on our toothpaste.*”

(Child 1, 4-year-old girl)

A few children also mentioned their habit of mouth rinsing as part of their oral hygiene routine. Although their descriptions were not extensive, they described the practice of using water and rinsing their mouth after eating. This habit served the purpose of clearing away any residual food that could potentially lead to dental problems. By swishing their mouth with water, children highlighted the importance of oral health care beyond toothbrushing.

“*After eating, we swish water in our mouth.*”

(Child 3, 4-year-old girl)

“*We do swish and swallow water.*”

(Child 2, 3-year-old girl)

#### 3.2.3. Toothbrush and Toothpaste Preferences

Toothbrush types such as manual or electric ones played a significant role in making toothbrushing interesting for young children. A few children explained their reasons for their preference for an electric toothbrush. Children enjoyed an electric toothbrush because of the playful vibrations and buzzing sounds. These electric toothbrushes transformed a routine task of toothbrushing into an exciting activity for children, as explained in quotes below:

All children: ”I like the electric toothbrush.” (at Preschool 1)

“*I like the electric one, I like that one, because it does on its own.*”

(Child 13, 4-year-old girl)

The majority of children showed preferences for a certain toothpaste mainly due to the captivating visual appeal of the packaging. The primary reason behind this was the presence of their favourite colour or familiar cartoon character adorning the toothpaste packaging, as described by preschool children in the quotes below:

“*I use Peppa Pig toothpaste.*”

(Child 3, 4-year-old girl)

“*I have a pink toothpaste.*”

(Child 1, 4-year-old girl)

### 3.3. Theme 3: Evaluation of Oral Health Promotion Kit

The BSBF oral health promotion program contributed to the children’s knowledge of oral health. While discussing their toothbrushing routine and understanding of the importance of toothbrushing, children mentioned that the oral health knowledge and skills they have are inherent, acquired unconsciously, or with/without direct instructions. The BSBF program aimed to educate children about the importance of maintaining good oral health and health-promoting oral health behaviours such as toothbrushing techniques, limiting sugar, and visiting a dentist. The BSBF oral health promotion involved showcasing positive oral health behaviours through a storytelling approach. Repetitive storytelling helped children add and retain knowledge to their implicit knowledge of good oral health. Children recalled and described the message of the story. The majority of the children liked the story and described the part or character of the story they liked most. A few children, however, disliked the story and wanted to change it to make it more interesting.

#### 3.3.1. Retention of Oral Health Messages

Children described the characters and outline of the story in the BSBF oral health promotion kit. They shared the knowledge they had acquired through the BSBF story and described the main theme of the story in the oral health promotion kit, indicating their engagement with the story content. They described the that they needed to visit a ‘doctor’, ‘nurse’, or ‘dentist’ to take care of their teeth. The BSBF activities not only helped to educate the children but also made learning about oral health care a fun and engaging experience for them.

“*We have to go and visit doctor for teeth.*”

(Child 3, 4-year-old girl)

“*We need to see dentist.*”

(Child 2, 3-year-old girl)

The majority of the children recounted a story that revolved around the significance of maintaining oral health and regular visits to the dentist. They mentioned that the story was about ‘cleaning the teeth’ and ‘going to the dentist’ by narrating it in a few words and stated that the BSBF story was informative for them. Although the children had some prior knowledge of the importance of toothbrushing, the story added to their understanding and fostered a positive association with oral health and dental check-ups.

“*The story was about cleaning teeth and going to a dentist. There was an alien there.*”

(Child 13, 4-year-old girl)

“*We brush each day, otherwise it turns into holes.*”

(Child 4, 4-year-old boy)

“*Alien come to Dr Rabbit, he wanted to know how to clean teeth.*”

(Child 8, 4-year-old boy)

#### 3.3.2. Feedback on Oral Health Promotion Kit

The BSBF program gained positive feedback from the majority of the children who participated in this study. While discussing the oral health promotion kit, the majority of the children admitted that they liked the story, especially the characters of ‘Dr Rabbit’ and ‘Scarlet’. Thus, the story included in the kit was enjoyable to children and contributed to an overall satisfying experience. One of the children assumed that Dr Rabbit and Scarlet were real characters and mentioned that they would like to play with them, as described in the quotes below:

“*I like to play with Scarlet and Dr Rabbit.*”

(Child 3, 4-year-old girl)

“*I like Dr Rabbit and learning about brushing your teeth.*”

(Child 5, 4-year-old girl)

Children often prefer to see familiar characters in the stories. Some children did not like the pictures in the story and wanted familiar cartoon characters or other characters in the storybook to make it more interesting, as described in the quotes below:

“*I do not like this because it does not have interesting pictures on it so I can’t read it and I don’t have it.*”

(Child 14, 4-year-old boy)

A few children did not like the story and suggested changing the story to make it engaging. Two children described clearly what they wanted to include in the story by providing character names such as Batman and Unicorn. However, there were a few children who were not able to provide any specific feedback on the story or the resource kit.

“*I want Batman in the story, Batman can come and then help Scarlet.*”

(Child 10, 4-year-old boy)

“*I want Unicorn in the story.*”

(Child 6, 3-year-old girl)

## 4. Discussion

The results from this study provide valuable insights into implementing the BSBF program in selected ECEC settings in New South Wales, Australia. This study was the first to identify children’s perceptions on implementing the BSBF oral health promotion program. Children expressed their perspectives on oral health in simple language and indicated that they understood the importance of routine oral healthcare habits to maintain healthy teeth and gums. Children consistently shared their understanding of the significance of oral health, aligning with the broader goals of oral health education and oral health promotion. The recognition of the importance of oral health underscores the BSBF program’s success in providing fundamental knowledge to early childhood learners.

Children demonstrated a commendable ability to remember key oral health messages, showcasing the program’s effectiveness in imparting short-term knowledge gain, consistent with the results of similar school-based oral health programs [51,52]. A study by Heilmann and colleagues [53] concluded that providing children with oral health knowledge during their early years laid the foundation for establishing lifelong practices, emphasizing the importance of early education in imparting lifelong oral health-promoting habits. Evidence suggests that health promotion in schools provides good outcomes, as the schools serve as ideal settings for implementing health promotion initiatives and can reach most children [54]. A systematic review by Bramantoro and colleagues [33] further concluded that school-based oral health promotion programs provided positive outcomes in enhancing oral health in children. Children who participated in this study highlighted the importance of oral hygiene practices to prevent tooth decay. This finding is consistent with the previous research [55] where children demonstrated a good understanding of oral health and the important role of oral health professionals in educating communities about oral health. Similar to other studies [55,56], the current study highlighted that preschool children, despite their young age, had a good understanding of the consequences of poor oral health behaviours [57,58]. These findings support the evidence that oral health promotion programs can shape young children’s perceptions of oral hygiene and effectively teach them how to achieve good oral health [17,22,23] that can lead to a better quality of life [59]. Children expressed their understanding of the importance of a healthy diet with low sugar and high intake of water in preventing dental caries, consistent with other studies examining the effect of oral health education [60,61]. Furthermore, the study by Khurshid [62] concluded that preventive oral health education may significantly reduce the incidence of dental caries in young children, as supported by the current study findings.

The role of parents is crucial in making oral hygiene practice a part of their children’s everyday routine. This study illustrates the importance of parents’ role in building children’s oral hygiene routine, which can include teaching them how to brush and floss their teeth and making oral care a fun activity, similar to the findings in previous research [63,64]. The study by Nepaul and Mahomed [65] supports the findings that parents’ oral health knowledge influences the oral health practices of children. Although not reported in our study, the study by Woodall et al. [66] reported that children disseminated oral health education information delivered at schools to their family members. The findings of our study shed light on the concept of situated learning, where parents act as role models in acquiring oral health skills, a finding consistent with previous research [55,66]. It is evident from earlier research [67,68,69] that the parent’s oral health influences the child’s oral health and, therefore, support from parents is critical for the long-term adoption of oral hygiene routines in children [70]. Several studies have reported that parents create supportive environments for children to prevent dental caries through providing nutritious meals, limiting sugary snacks/drinks, observing the frequency of toothbrushing, demonstrating proper oral hygiene techniques, and actively engaging in oral health discussions with children [71,72]. It is imperative to emphasize the pivotal role of parents and caregivers in shaping children’s health-promoting habits. Despite preschool-aged children potentially grasping some aspects of these habits, the fundamental responsibility lies with adults to offer guidance, consistent support, and ongoing reinforcement of the significance of oral health habits.

In the current study, children expressed their preferences towards the taste of toothpaste. It is interesting to note that preference for different types of taste (e.g., sweet, bitter, salty, sour) is known to differ between children of different age groups [73,74]. From birth, children have a preference for sweet taste, reject sour taste, and are indifferent to salty and bitter tastes [73,74]. However, taste preferences change with time, and preschool children have a higher preference for sweet, salty, and sour tastes, and reject bitter tastes, which typically lasts until late adolescence (65–67). Previous research highlighted that the flavour of children’s toothpaste may encourage the development of good oral health habits [75]. Studies on toothbrushing practices support the association between toothpaste flavours and increased brushing time in children, which may lead to the increased efficacy of fluoride in toothpaste [76]. Nonetheless, the use of flavoured toothpaste for children has received great attention in the last three decades due to concerns over ingestion and the risk of dental fluorosis and fluoride toxicity [77]. This study also highlighted the potential impact of engaging tools and packaging on encouraging better oral hygiene practices, similar to factors documented in previous research [78,79]. The study by Choudhari et al. [78] concluded that if toothpaste is designed with the characteristics of children’s preferences, it can help to motivate children to brush and maintain good oral health. In addition to the toothpaste, children expressed their preferences for electric and manual toothbrushes. The study by Karimi [80] compared the advantages of both these types of toothbrushes and concluded that children’s preference for electric toothbrushes was due to their features such as automation, speed adjustment, LCD screen, and making toothbrushing a fun activity. Evidence from a Cochrane Systematic Review further highlights that powered toothbrushes may reduce dental plaque and gingivitis in the short term [81].

The evaluation of the BSBF oral health promotion kit demonstrated positive feedback for children’s retention of oral health messages and overall satisfaction. The BSBF program used a storytelling method involving characters such as ‘Dr Rabbit’ and ‘Scarlet,’ who effectively conveyed key oral health messages, similar to factors documented in previous research [23]. Preschool children in this study remembered the key themes of the story and expressed their liking for the captivating characters. However, a few children expressed their dislike for some characters and indicated their preferences for familiar characters such as Batman or Unicorn, noting a potential scope for improvement in tailoring characters to align with the interests of the children. These findings are supported by evidence suggesting that children’s preferences for specific characteristics in oral health materials may act as a potential barrier to implementing toothbrushing programs [79]. The feedback provided by children emphasises the significance of incorporating elements that connect with children, providing a more enjoyable and engaging learning experience, a finding consistent with the previous research [82]. The study by Malik et al. [83] further concluded that utilising engaging characters, games, and activities aimed at the developmental level of young children was a more effective method to deliver oral health messages. Other researchers have also noted that storytelling can be a useful tool for disseminating and communicating evidence in healthcare in general [84] and in oral health promotion for preschool children [85]. Furthermore, systematic reviews have found that changing health behaviours using storytelling is promising, as stories help people identify with others, and, in turn, reduce resistance and inspire new health behaviours [84,86].

Several studies have emphasized the critical role of children’s active participation and engagement in oral health promotion programs [79,87]. The recent systematic review by Chandio and colleagues [34] further reported that children’s engagement with and acceptability of programs, and a continuation of a similar practice at home, were key enablers among children for implementing oral health promotion programs. The review by Quach [35] and a systematic review by Hakojarvi and colleagues [30] reported that children are primarily involved in studies during the implementation phase of oral health promotion interventions but not during the evaluation phase and, therefore, studies with participatory research methodology are required to gain a better understanding of children perceptive about the intervention. Our study addresses this knowledge gap as we obtained young children’s feedback during the evaluation of the BSBF oral health promotion program. Future studies should focus on assessing the sustainability of oral health promotion programs, considering factors such as the availability and accessibility of oral health resources and social-environmental determinants of child oral health [88].

### Strengths and Limitations

This study has several strengths and limitations. Employing focus groups to facilitate open expression and discussion of children’s perspectives on the BSBF oral health promotion program is a strength. The inclusion of preschool children to gain their views and insights is the key strength, as young children’s views are often overlooked in health research. Data were collected through three focus groups with a total sample of 15 children. Although the literature suggests that two to three focus groups are enough to reach data saturation in qualitative research [46], it is possible that our findings may not allow for generalisation to other geographical settings. Further, social desirability bias may have influenced responses from children leading to an overemphasis on positive oral health behaviours. Educators were present during focus group discussions, potentially impacting the children’s responses positively or negatively. Despite the limitations, this study’s focus on diverse perspectives provides valuable insights into the experiences and perceptions of the BSBF oral health promotion program.

## 5. Conclusions

In conclusion, this qualitative study provides valuable insights into how the BSBF oral health promotion program influences preschool children. The findings of the study emphasise the success of the BSBF program in improving oral health knowledge, retaining key oral health messages, shaping positive attitudes towards oral health, and making oral hygiene part of daily routine. Parents and caregivers act as role models for young children and, therefore, family-centered approaches to prevent tooth decay and maintain oral health are critical. Whilst it is important for researchers and policy makers to actively involve young children in oral health promotion, it is imperative to emphasize the pivotal role of educators, caregivers, and parents in shaping children’s understanding of oral health. 

## Figures and Tables

**Table 1:** Themes and subthemes emerged from the three focus group discussions.

Themes	Subthemes
Theme 1: Oral health knowledge	Importance of oral health Preventing tooth decay
Theme 2: Oral hygiene practices	Making oral hygiene part of daily routine and role of parents Steps to follow in toothbrushing practices Toothbrush and toothpaste preferences
Theme 3: Evaluation of oral health promotion kit	Retention of oral health messages Feedback on oral health promotion kit

## Data Availability

Data are contained within the article or Supplementary Material. The data presented in this study are available in the Results Section.

## Supplementary Materials

The following supporting information can be downloaded at: https://www.mdpi.com/article/10.3390/children11040415/s1. Table S1: COREQ (Consolidated Criteria for Reporting Qualitative Research) Checklist.

## References

[1] Ha D., Roberts-Thomson K., Peres K., Arrow P., Do L., Do L., Spencer J.A. (2016). Oral health status of Australian children. Oral Health of Australian Children: The National Child Oral Health Study 2012–14.

[2] Thomson W.M. (2016). Public health aspects of paediatric dental treatment under general anaesthetic. Dent. J..

[3] Casamassimo P.S., Thikkurissy S., Edelstein B.L., Maiorini E. (2009). Beyond the dmft: The human and economic cost of early childhood caries. J. Am. Dent. Assoc..

[4] Australian Institute of Health and Welfare (2020). Oral Health and Dental Care in Australia.

[5] Do L., Spencer A. (2016). Oral Health of Australian Children: The National Child Oral Health Study 2012–14.

[6] James S.L., Abate D., Abate K.H., Abay S.M., Abbafati C., Abbasi N., Abbastabar H., Abd-Allah F., Abdela J., Abdelalim A. (2018). Global, regional, and national incidence, prevalence, and years lived with disability for 354 diseases and injuries for 195 countries and territories, 1990–2017: A systematic analysis for the Global Burden of Disease Study 2017. Lancet.

[7] Singh N., Dubey N., Rathore M., Pandey P. (2020). Impact of early childhood caries on quality of life: Child and parent perspectives. J. Oral Biol. Craniofacial Res..

[8] Tinanoff N., Baez R.J., Diaz Guillory C., Donly K.J., Feldens C.A., McGrath C., Phantumvanit P., Pitts N.B., Seow W.K., Sharkov N. (2019). Early childhood caries epidemiology, aetiology, risk assessment, societal burden, management, education, and policy: Global perspective. Int. J. Paediatr. Dent..

[9] Zaror C., Matamala-Santander A., Ferrer M., Rivera-Mendoza F., Espinoza-Espinoza G., Martínez-Zapata M.J. (2022). Impact of early childhood caries on oral health-related quality of life: A systematic review and meta-analysis. Int. J. Dent. Hyg..

[10] American Academy of Pediatric Dentistry (2016). Policy on Early Childhood Caries (ECC): Classifications, Consequences, and Preventive Strategies. Pediatr. Dent..

[11] Kirthiga M., Murugan M., Saikia A., Kirubakaran R. (2019). Risk factors for early childhood caries: A systematic review and meta-analysis of case control and cohort studies. Pediatr. Dent..

[12] Arora A., Schwarz E., Blinkhorn A.S. (2011). Risk factors for early childhood caries in disadvantaged populations. J. Investig. Clin. Dent..

[13] Balakrishnan M., Simmonds R.S., Tagg J.R. (2000). Dental caries is a preventable infectious disease. Aust. Dent. J..

[14] Sicca C., Bobbio E., Quartuccio N., Nicolò G., Cistaro A. (2016). Prevention of dental caries: A review of effective treatments. J. Clin. Exp. Dent..

[15] Welti R., Jones B., Moynihan P., Silva M. (2023). Evidence pertaining to modifiable risk factors for oral diseases: An umbrella review to Inform oral health messages for Australia. Aust. Dent. J..

[16] Rana K., Ekanayake K., Chimoriya R., Palu E., Do L., Silva M., Tadakamadla S., Bhole S., Leshargie C., Wen L. (2022). Effectiveness of Oral Health Promotion Interventions: An Evidence Check Rapid Review.

[17] Tahani B., Asgari I., Golkar S., Ghorani A., Hasan N., Tehrani Z., Moghadam F.A. (2022). Effectiveness of an integrated model of oral health-promoting schools in improving children’s knowledge and the KAP of their parents, Iran. BMC Oral Health.

[18] World Health Organization School and Youth Health: Global School Health Initiative. https://www.who.int/health-topics/health-promoting-schools#tab=tab_1.

[19] Kwan S.Y., Petersen P.E., Pine C.M., Borutta A. (2005). Health-promoting schools: An opportunity for oral health promotion. Bull. World Health Organ..

[20] Keshavarz N., Nutbeam D., Rowling L., Khavarpour F. (2010). Schools as social complex adaptive systems: A new way to understand the challenges of introducing the health promoting schools concept. Soc. Sci. Med..

[21] World Health Organization WHO Information Series on School Health—Oral Health Promotion: An Essential Element of a Health-Promoting School. https://www3.paho.org/hq/dmdocuments/2009/OH-st-sch.pdf.

[22] Dickson-Swift V., Kenny A., Gussy M., McCarthy C., Bracksley-O’Grady S. (2020). The knowledge and practice of pediatricians in children’s oral health: A scoping review. BMC Oral Health.

[23] Office of Oral Health Growing Healthy Smiles in the Child Care Setting. https://www.mass.gov/doc/growing-healthy-smiles-in-the-child-care-setting-2/download.

[24] Dickson-Swift V., Kenny A., Gussy M., de Silva A.M., Farmer J., Bracksley-O’Grady S. (2017). Supervised toothbrushing programs in primary schools and early childhood settings: A scoping review. Community Dent. Health.

[25] Fraihat N., Madae’en S., Bencze Z., Herczeg A., Varga O. (2019). Clinical Effectiveness and Cost-Effectiveness of Oral-Health Pro-motion in Dental Caries Prevention among Children: Systematic Review and Meta-Analysis. Int. J. Environ. Res. Public Health.

[26] Wong N.T., Zimmerman M.A., Parker E.A. (2010). A typology of youth participation and empowerment for child and adolescent health promotion. Am. J. Community Psychol..

[27] Harcourt D., Einarsdottir J. (2011). Introducing children’s perspectives and participation in research. Eur. Early Child. Educ. Res. J..

[28] Statham J., Chase E. (2010). Childhood Wellbeing: A Brief Overview.

[29] Marshman Z., Gupta E., Baker S.R., Robinson P.G., Owens J., Rodd H.D., Benson P.E., Gibson B. (2015). Seen and heard: Towards child participation in dental research. Int. J. Paediatr. Dent..

[30] Hakojärvi H.R., Selänne L., Salanterä S. (2019). Child involvement in oral health education interventions—A systematic review of randomised controlled studies. Community Dent. Health.

[31] Ghaffari M., Rakhshanderou S., Ramezankhani A., Buunk-Werkhoven Y., Noroozi M., Armoon B. (2018). Are educating and promoting interventions effective in oral health?: A systematic review. Int. J. Dent. Hyg..

[32] Tsai C., Raphael S., Agnew C., McDonald G., Irving M. (2020). Health promotion interventions to improve oral health of adolescents: A systematic review and meta-analysis. Community Dent. Oral Epidemiol..

[33] Bramantoro T., Santoso C.M.A., Hariyani N., Setyowati D., Zulfiana A.A., Nor N.A.M., Nagy A., Pratamawari D.N.P., Irmalia W.R. (2021). Effectiveness of the school-based oral health promotion programmes from preschool to high school: A systematic review. PLoS ONE.

[34] Chandio N., Micheal S., Tadakmadla S.K., Sohn W., Cartwright S., White R., Sanagavarapu P., Parmar J.S., Arora A. (2022). Barriers and enablers in the implementation and sustainability of toothbrushing programs in early childhood settings and primary schools: A systematic review. BMC Oral Health.

[35] Quach H. (2020). How can children be involved in developing oral health education interventions?. Evid.-Based Dent..

[36] Colgate-Palmolive Bright Smiles, Bright Futures (BSBF). https://www.colgatepalmolive.com/en-us/community-impact/bright-smiles-bright-futures.

[37] Ritchie J., Lewis J., McNaughton Nicholls C., Ormston R. (2014). Qualitative Research Practice a Guide for Social Science Students and Researchers.

[38] Tong A., Sainsbury P., Craig J. (2007). Consolidated criteria for reporting qualitative research (COREQ): A 32-item checklist for interviews and focus groups. Int. J. Qual. Health Care.

[39] Dupper D.R., Forrest-Bank S., Lowry-Carusillo A. (2015). Experiences of religious minorities in public school settings: Findings from focus groups involving Muslim, Jewish, Catholic, and Unitarian Universalist youths. Child. Sch..

[40] Adler K., Salanterä S., Zumstein-Shaha M. (2019). Focus group interviews in child, youth, and parent research: An integrative literature review. Int. J. Qual. Methods.

[41] Cammisa M., Montrone R., Caroli M. (2011). Development and results of a new methodology to perform focus group with preschool children on their beliefs and attitudes on physical activity. Int. J. Pediatr. Obes..

[42] Koller D., San Juan V. (2015). Play-based interview methods for exploring young children’s perspectives on inclusion. Int. J. Qual. Stud. Educ..

[43] Wong L.P. (2008). Focus group discussion: A tool for health and medical research. Singap. Med. J..

[44] Harcourt D., Conroy H. (2011). Informed consent: Processes and procedures in seeking research partnerships with young children. Researching Young Children’s Perspectives.

[45] Heary C.M., Hennessy E. (2002). The use of focus group interviews in pediatric health care research. J. Pediatr. Psychol..

[46] Guest G., MacQueen K.M., Namey E.E. (2011). Applied Thematic Analysis.

[47] Braun V., Clarke V. (2006). Using thematic analysis in psychology. Qual. Res. Psychol..

[48] Antin T.M.J., Constantine N.A., Hunt G. (2014). Conflicting Discourses in Qualitative Research: The Search for Divergent Data within Cases. Field Methods.

[49] Johnson J.L., Adkins D., Chauvin S. (2020). A Review of the Quality Indicators of Rigor in Qualitative Research. Am. J. Pharm. Educ..

[50] Holmes A. (2020). Researcher Positionality—A Consideration of Its Influence and Place in Qualitative Research—A New Researcher Guide. Shanlax Int. J. Educ..

[51] Al Bardaweel S., Dashash M. (2018). E-learning or educational leaflet: Does it make a difference in oral health promotion? A clustered randomized trial. BMC Oral Health.

[52] Pradhan D., Pruthi N., Sharma L., Chavan J., Verma P. (2020). Impact of oral health education on oral health knowledge, attitude, and practices among 13–15 years’ school-going children from Kanpur city, India: A quasi-experimental study. J. Indian Assoc. Public Health Dent..

[53] Heilmann A., Tsakos G., Watt R.G. (2015). Oral health over the life course. A Life Course Perspect. Health Trajectories Transit..

[54] WHO WHO Guideline on School Health Services. https://www.who.int/publications/i/item/9789240029392.

[55] Davies K., Lin Y.L., Callery P. (2017). Parents’ and children’s knowledge of oral health: A qualitative study of children with cleft palate. Int. J. Paediatr. Dent..

[56] Vishwanathaiah S. (2016). Knowledge, Attitudes, and Oral Health Practices of School Children in Davangere. Int. J. Clin. Pediatr. Dent..

[57] Melo P., Fine C., Malone S., Frencken J.E., Horn V. (2018). The effectiveness of the Brush Day and Night programme in improving children’s toothbrushing knowledge and behaviour. Int. Dent. J..

[58] Zeeberg C., Perez Puello S.d.C., Batista M.J., Sousa M.d.L.R.d. (2018). Effectiveness of a preventive oral health program in preschool children. Braz. J. Oral Sci..

[59] Han J., Park H. (2017). Effect of Oral Health Education on Oral Health Knowledge, Oral Health Behavior and Oral Hygiene Status in Children from North Korea. Child Health Nurs. Res..

[60] Naidu R.S., Nunn J.H. (2020). Oral health knowledge, attitudes and behaviour of parents and caregivers of preschool children: Implications for oral health promotion. Oral Health Prev. Dent..

[61] Al-Qahtani S.M., Razak P.A., Khan S.D. (2020). Knowledge and Practice of Preventive Measures for Oral Health Care among Male Intermediate Schoolchildren in Abha, Saudi Arabia. Int. J. Environ. Res. Public Health.

[62] Shen A., Bernabé E., Sabbah W. (2021). Systematic Review of Intervention Studies Aiming at Reducing Inequality in Dental Caries among Children. Int. J. Environ. Res. Public Health.

[63] Dhull K.S., Dutta B., Devraj I.M., Samir P.V. (2018). Knowledge, Attitude, and Practice of Mothers towards Infant Oral Healthcare. Int. J. Clin. Pediatr. Dent..

[64] de Silva-Sanigorski A., Ashbolt R., Green J., Calache H., Keith B., Riggs E., Waters E. (2013). Parental self-efficacy and oral health-related knowledge are associated with parent and child oral health behaviors and self-reported oral health status. Community Dent. Oral Epidemiol..

[65] Nepaul P., Mahomed O. (2020). Influence of parents’ oral health knowledge and attitudes on oral health practices of children (5–12 years) in a rural school in KwaZulu-Natal, South Africa. J. Int. Soc. Prev. Community Dent..

[66] Woodall J., Woodward J., Witty K., McCulloch S. (2014). An evaluation of a toothbrushing programme in schools. Health Educ..

[67] Moin M., Saadat S., Rafique S., Maqsood A., Lal A., Vohra F., Alam M.K., Ahmed N. (2021). Impact of Oral Health Educational Interventions on Oral Hygiene Status of Children with Hearing Loss: A Randomized Controlled Trial. BioMed Res. Int..

[68] Knipe D., Evans H., Marchant A., Gunnell D., John A. (2020). Mapping population mental health concerns related to COVID-19 and the consequences of physical distancing: A Google trends analysis. Wellcome Open Res..

[69] Chen L., Hong J., Xiong D., Zhang L., Li Y., Huang S., Hua F. (2020). Are parents’ education levels associated with either their oral health knowledge or their children’s oral health behaviors? A survey of 8446 families in Wuhan. BMC Oral Health.

[70] Gray-Burrows K.A., Day P.F., Marshman Z., Aliakbari E., Prady S.L., McEachan R.R.C. (2016). Using intervention mapping to develop a home-based parental-supervised toothbrushing intervention for young children. Implement. Sci..

[71] Varkey I.M., Ghule K.D., Mathew R., Desai J., Gomes S., Mudaliar A., Bhori M., Tungare K., Gharat A. (2022). Assessment of attitudes and practices regarding oral healthcare during the COVID-19 pandemic among the parents of children aged 4–7 years. Dent. Med. Probl..

[72] Nation A., Pukallus M., Stormon N., Foley M., Lalloo R. (2023). Health professionals delivering oral health interventions in early childhood: A scoping review of Australian and New Zealand literature. Health Promot. J. Aust..

[73] Beauchamp G.K., Mennella J.A. (2011). Flavor Perception in Human Infants: Development and Functional Significance. Digestion.

[74] Mennella J.A., Pepino M.Y., Reed D.R. (2005). Genetic and environmental determinants of bitter perception and sweet preferences. Pediatrics.

[75] Stovell A.G., Newton B.M., Lynch R.J.M. (2020). Important considerations in the development of toothpaste formulations for children. Int. Dent. J..

[76] Newby E.E., Martinez-Mier E.A., Zero D.T., Kelly S.A., Fleming N., North M., Bosma M.L. (2013). A randomised clinical study to evaluate the effect of brushing duration on fluoride levels in dental biofilm fluid and saliva in children aged 4–5 years. Int. Dent. J..

[77] Kobayashi C.A.N., Belini M.R., Italiani F.d.M., Pauleto A.R.C., Julianelli de Araújo J., Tessarolli V., Grizzo L.T., Pessan J.P., Machado M.A.d.A.M., Buzalaf M.A.R. (2011). Factors influencing fluoride ingestion from dentifrice by children. Community Dent. Oral Epidemiol..

[78] Choudhari S., Gurunathan D., Kanthaswamy A. (2020). Children’s perspective on color, smell and flavor of toothpaste. Indian J. Dent. Res..

[79] Dimitropoulos Y., Gunasekera H., Blinkhorn A., Byun R., Binge N., Gwynne K., Irving M. (2018). A collaboration with local Aboriginal communities in rural New South Wales, Australia to determine the oral health needs of their children and develop a community-owned oral health promotion program. Rural Remote Health.

[80] Karimi M. (2021). What’s Better for Children; an Electric or Manual Toothbrush?. Interv. Pediatr. Dent. Open Access J..

[81] Yaacob M., Worthington H.V., Deacon S.A., Deery C., Walmsley A.D., Robinson P.G., Glenny A.M., Worthington H.V. (2014). Powered versus manual toothbrushing for oral health. Cochrane Database Syst. Rev..

[82] Heaton B., Gebel C., Crawford A., Barker J.C., Henshaw M., Garcia R.I., Riedy C., Wimsatt M.A. (2018). Using Storytelling to Address Oral Health Knowledge in American Indian and Alaska Native Communities. Prev. Chronic Dis..

[83] Malik A., Sabharwal S., Kumar A., Singh Samant P., Singh A., Kumar Pandey V. (2017). Implementation of Game-based Oral Health Education vs Conventional Oral Health Education on Children’s Oral Health-related Knowledge and Oral Hygiene Status. Int. J. Clin. Pediatr. Dent..

[84] Rose R., Chakraborty S., Mason-Lai P., Brocke W., Page S.A., Cawthorpe D. (2016). The storied mind: A meta-narrative review exploring the capacity of stories to foster humanism in health care. J. Hosp. Adm..

[85] Shruti T., Govindraju H.A., Sriranga J. (2021). Incorporation of Storytelling as a Method of Oral Health Education among 3–6-year-old Preschool Children. Int. J. Clin. Pediatr. Dent..

[86] Perrier M.-J., Martin Ginis K.A. (2018). Changing health-promoting behaviours through narrative interventions: A systematic review. J. Health Psychol..

[87] Elsadek Y.E.E., Baker S.R.R. (2023). Oral health promotion through health-promoting schools in developing countries: A scoping review. Community Dent. Oral Epidemiol..

[88] Quintero Valencia C.A., Robledo Bermúdez D.P., Vásquez Hernández A., Delgado Restrepo O., Franco Cortés Á.M. (2014). Barriers to dental care access during early childhood. Medellín, 2007. Rev. Fac. Odontol. Univ. Antioq..

