# Supplementary material for: A Qualitative Study of Preschool Children’s Perspectives on an Oral Health Promotion Program in New South Wales, Australia
Source: Children (Basel). 2024 Apr 1;11(4):415. doi: 10.3390/children11040415 (PMC11049284; doi:10.3390/children11040415)

Supplementary Material

Table S1. COREQ (COnsolidated criteria for REporting Qualitative research) Checklist.

Item No. and Topic	Guide Questions/Description	Response and Reported on Page No.
**Domain 1: Research team and reflexivity**		
Personal characteristics		
1. Interviewer/facilitator	Which author/s conducted the interview or focus group?	P.S, A.A., S.M.
2. Credentials	What were the researcher's credentials? E.g. PhD, MD	P.S.: PhD<br>A.A.: PhD<br>S.M.: PhD
3. Occupation	What was their occupation at the time of the study?	Researchers
4. Gender	Was the researcher male or female?	Two female and one male
5. Experience and training	What experience or training did the researcher have?	Interviewers had prior experience in qualitative interviewing, early childhood education and child oral health.
Relationship with participants		
6. Relationship established	Was a relationship established prior to study commencement?	Researchers had no relationships with the participants before the study.
7. Participant knowledge of the interviewer	What did the participants know about the researcher? e.g. personal goals, reasons for doing the research	Parents of the participants knew about the reasons for doing research as indicated in the participant information sheets. Children were explained about the research before the commencement of the focus group.
8. Interviewer characteristics	What characteristics were reported about the inter viewer/facilitator? e.g. Bias, assumptions, reasons and interests in the research topic	Apart from their names and affiliation, participants did not know about interviewers' characteristics.
**Domain 2: Study design**		
Theoretical framework		
9. Methodological orientation and Theory	What methodological orientation was stated to underpin the study? e.g. grounded theory, discourse analysis, ethnography, phenomenology, content analysis	Qualitative Research Design
Participant selection		
10. Sampling	How were participants selected? e.g. purposive, convenience, consecutive, snowball	Convenience sampling
11. Method of approach	How were participants approached? e.g. face-to-face, telephone, mail, email	Invitation from preschools
12. Sample size	How many participants were in the study?	15 children
13. Non-participation	How many people refused to participate or dropped out? Reasons?	None refused to participate or dropped out
Setting		
14. Setting of data collection	Where was the data collected? e.g. home, clinic, workplace	Early Childhood Education and Care settlings
15. Presence of non-participants	Was anyone else present besides the participants and researchers?	Preschool Educatoe
16. Description of sample	What are the important characteristics of the sample? e.g. demographic data, date	The total sample included 7 boys and 8 girls, aged 3-5 years.
Data collection		
17. Interview guide	Were questions, prompts, guides provided by the authors? Was it pilot tested?	Semi-structured interview guide. It was also pilot tested
18. Repeat interviews	Were repeat interviews carried out? If yes, how many?	Repeat interviews were not carried out

19. Audio/visual recording	Did the research use audio or visual recording to collect the data?	Interviews were audio-recorded (
20. Field notes	Were field notes made during and/or after the interview or focus group?	Field notes were made immediately after each interview and they helped in data analysis.
21. Duration	What was the duration of the interviews or focus group?	20-30 minutes
22. Data saturation	Was data saturation discussed?	Yes
23. Transcripts returned	Were transcripts returned to participants for comment and/or correction?	No
**Domain 3: Analysis and findings**		
Data analysis		
24. Number of data coders	How many data coders coded the data?	3 researchers- J.P., A.A., P.S.
25. Description of the coding tree	Did authors provide a description of the coding tree?	Yes
26. Derivation of themes	Were themes identified in advance or derived from the data?	Themes were derived from the data
27. Software	What software, if applicable, was used to manage the data?	Quirkos software
28. Participant checking	Did participants provide feedback on the findings?	No
Reporting		
29. Quotations presented	Were participant quotations presented to illustrate the themes/findings? Was each quotation identified? e.g. participant number	Yes, participant quotations were presented. Quotations were de-identified for confidentiality reasons.
30. Data and findings consistent	Was there consistency between the data presented and the findings?	Yes, as indicated in Rigor
31. Clarity of major themes	Were major themes clearly presented in the findings?	Yes, presented in Results
32. Clarity of minor themes	Is there a description of diverse cases or discussion of minor themes?	Yes, presented in Discussion

Developed from: Tong A, Sainsbury P, Craig J. Consolidated criteria for reporting qualitative research (COREQ): a 32-item checklist for interviews and focus groups. *International Journal for Quality in Health Care*. 2007. Volume 19, Number 6: pp. 349 – 357

Figure S1. Thematic map showing the interaction between themes and subthemes.

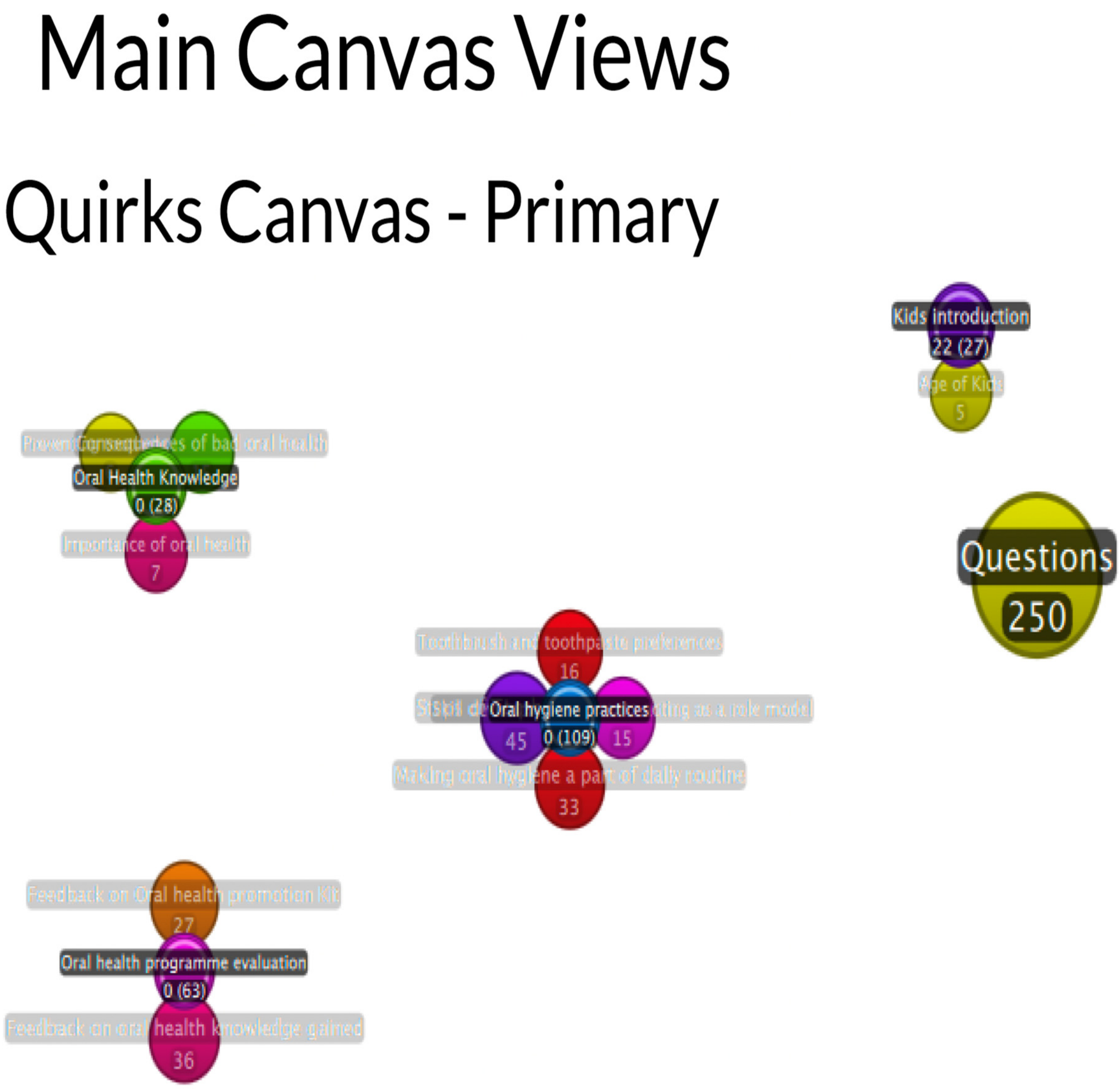

Supplement: Supplementary file 1 [file children-11-00415-s001.zip › children-2901671-supplementary.pdf]
